# O04 Reintroducing pen and leaving quin-alone: examining the impact of a penicillin allergy de-labelling project on antibiotic prescribing in a hospital setting

**DOI:** 10.1093/jacamr/dlad143.004

**Published:** 2024-01-03

**Authors:** Lisa Duffy, Jennifer Mulhall, Deirdre Cunningham, Cathleen Moran, Alan Connelly, Mary O’Malley, Ksenia Davenport, Caríosa Lee-Brennan, David Gallagher

**Affiliations:** Department of Infectious Disease, University Hospital Galway, Galway, Ireland; Department of Immunology, University Hospital Galway, Galway, Ireland; Pharmacy Department, University Hospital Galway, Galway, Ireland; Acute Medical Unit, University Hospital Galway, Galway, Ireland; Acute Medical Unit, University Hospital Galway, Galway, Ireland; Acute Medical Unit, University Hospital Galway, Galway, Ireland; Department of Infectious Disease, University Hospital Galway, Galway, Ireland; Department of Immunology, University Hospital Galway, Galway, Ireland; Department of Infectious Disease, University Hospital Galway, Galway, Ireland

## Abstract

**Background:**

The label of penicillin allergy is carried by between 8 and 25% of all patients (1). Alternative antibiotics may not be as efficacious, can be more expensive and lead to more adverse effects (2). Interrogation of a penicillin allergy is an important stewardship intervention considering only 1-10% of these patients are truly allergic to penicillin (1). The aim of this study was to investigate if a penicillin allergy de-labelling project altered antibiotics subsequently prescribed in a hospital setting.

**Methods:**

A review was carried out of 75 patients identified as part of a penicillin allergy de-labelling project in Galway University Hospital over a 15 month period. Self-reported penicillin allergy by hospitalized patients was investigated and clarified by a collaboration from pharmacy, immunology and infectious diseases. Allergy labels were either reinforced, immediately de-labelled or further investigated with skin testing or oral challenge. Antibiotics prescribed prior to allergy de-labelling and following allergy clarification were noted. This information was obtained from the electronic hospital record.

**Results:**

In total, 118 patients had penicillin allergy assessment. 75 patients had their penicillin allergy de-labelled. 44 patients were de-labelled from history, 22 were de-labelled after skin testing and oral challenge and 9 were de-labelled following an oral challenge only. 47 patients (63%) received penicillin post de-labelling. Out of these 47 patients, 27 had been previously treated with fluoroquinolones (57%), 4 had received teicoplanin surgical prophylaxis for surgical procedures (9%), 4 had received linezolid, 2 had received ceftazidime and 2 had been seemingly prescribed meropenem based on allergy.

**Conclusions:**

These results demonstrate that appropriate de-labelling of penicillin allergy has a positive impact on future antibiotic prescription in a hospital setting. It results in reduced use of fluoroquinolones as well as other restricted antibiotics with higher risk of adverse effects and is a useful antimicrobial stewardship tool.
